# Pre-hospital cooling of patients following cardiac arrest is effective using even low volumes of cold saline

**DOI:** 10.1186/cc9386

**Published:** 2010-12-22

**Authors:** Roman Škulec, Anatolij Truhlář, Jana Šeblová, Pavel Dostál, Vladimír Černý

**Affiliations:** 1 Emergency Medical Service of the Central Bohemian Region, Prof. Veseleho 461, Beroun 266 01, Czech Republic; 2 Department of Anesthesiology and Intensive Care, Charles University in Prague, Faculty of Medicine in Hradec Kralove, University Hospital Hradec Kralove, Hradec Kralove 500 05, Czech Republic; 3 Beroun City Hospital, Jessenia a.s., Prof. Veseleho 451, Beroun 266 01, Czech Republic; 4 Hradec Kralove Region Emergency Medical Services, Hradecka 1690/2A, Hradec Kralove 500 12, Czech Republic; 5 Emergency Medical Service of the Central Bohemian Region, Vančurova 1544, Kladno 272 01, Czech Republic; 6 Department of Anesthesia, Dalhousie University, 1276 South Park Street, 10 West, Victoria Building, Halifax, NS, B3H 2Y9, Canada

## Abstract

**Introduction:**

Pre-hospital induction of therapeutic mild hypothermia (TH) may reduce post-cardiac arrest brain injury in patients resuscitated from out-of-hospital cardiac arrest. Most often, it is induced by a rapid intravenous administration of as much as 30 ml/kg of cold crystalloids. We decided to assess the pre-hospital cooling effectivity of this approach by using a target dose of 15-20 ml/kg of 4°C cold normal saline in the setting of the physician-staffed Emergency Medical Service. The safety and impact on the clinical outcome have also been analyzed.

**Methods:**

We performed a prospective observational study with a retrospective control group. A total of 40 patients were cooled by an intravenous administration of 15-20 ml/kg of 4°C cold normal saline during transport to the hospital (TH group). The pre-hospital decrease of tympanic temperature (TT) was analyzed as the primary endpoint. Patients in the control group did not undergo any pre-hospital cooling.

**Results:**

In the TH group, administration of 12.6 ± 6.4 ml/kg of 4°C cold normal saline was followed by a pre-hospital decrease of TT of 1.4 ± 0.8°C in 42.8 ± 19.6 min (p < 0.001). The most effective cooling was associated with a transport time duration of 38-60 min and with an infusion of 17 ml/kg of cold saline. In the TH group, a trend toward a reduced need for catecholamines during transport was detected (35.0 vs. 52.5%, p = 0.115). There were no differences in demographic variables, comorbidities, parameters of the cardiopulmonary resuscitation and in other post-resuscitation characteristics. The coupling of pre-hospital cooling with subsequent in-hospital TH predicted a favorable neurological outcome at hospital discharge (OR 4.1, CI95% 1.1-18.2, p = 0.046).

**Conclusions:**

Pre-hospital induction of TH by the rapid intravenous administration of cold normal saline has been shown to be efficient even with a lower dose of coolant than reported in previous studies. This dose can be associated with a favorable impact on circulatory stability early after the return of spontaneous circulation and, when coupled with in-hospital continuation of cooling, can potentially improve the prognosis of patients.

**Trial Registration:**

ClinicalTrials (NCT): NCT00915421

## Introduction

Therapeutic mild hypothermia (TH) has become a routine part of in-hospital post-resuscitation support. It has been recommended that the target therapeutic temperature be reached as soon as possible [1]. Thus, in successfully resuscitated out-of-hospital cardiac arrest (OHCA) patients, pre-hospital initiation of cooling appears to be a method of choice. A few studies demonstrating the efficacy and safety of this strategy, predominantly for the technique of rapid intravenous administration of cold crystalloids (RIVA), have been published [2-6]. In general, a target dose of 30 mL/kg was recommended. However, this dose is not easy to reach in routine practice, especially when transport time is short. Therefore, we performed a clinical study to assess a pre-hospital cooling effectivity of RIVA with the target dose of 15 to 20 mL/kg of 4°C cold normal saline in the setting of the physician-staffed emergency medical service (EMS). The safety and impact on the clinical outcome have also been analyzed.

## Materials and methods

We performed a multicenter prospective observational study with a retrospective control group in 18 physician-staffed bases of the EMS and in 23 intensive care units (ICUs) of two administrative regions of the Czech Republic (tributary area of 1,840,000 inhabitants). The study was conducted in accordance with the Declaration of Helsinki; was approved by the ethics committee of University Hospital Hradec Kralove, by the Czech Society for Emergency and Disaster Medicine, the Czech Society of Anaesthesiology and Intensive Care Medicine, and the Czech Society of Intensive Care Medicine; and was endorsed by the Czech Clinical Trial Network of the Czech Society of Anaesthesiology and Intensive Care Medicine and the Czech Society of Intensive Care Medicine. The study was named PRE-COOL (Pre-Hospital Cooling in Cardiac Arrest Patients). Because the study was non-randomized and no new drug, therapeutic procedure, or diagnostic procedure was evaluated, written informed consent was not required.

Patients meeting the inclusion and exclusion criteria were included in the prospective group with active cooling (TH group). The inclusion criterion was successfully resuscitated OHCA with any initial rhythm, persistence of coma, and requirement of mechanical ventilation. Exclusion criteria were cardiac arrest of traumatic origin, patient conscious after short cardiopulmonary resuscitation (CPR), coma of origin other than cardiac arrest, acute pulmonary edema, active severe bleeding [7], circulatory shock defined as hypotension irresponsive to volume expansion or vasopressoric support or both, severe bradycardia requiring transcutaneous cardiac pacing, severe sepsis/septic shock [8], pregnancy, and a do-not-resuscitate or do-not-intubate status. CPR was performed in accordance with European Resuscitation Council guidelines [1]. After the return of spontaneous circulation (ROSC), the initial assessment of vital signs, including 12-lead electrocardiogram and body temperature measurement, was performed. Then patients had additional intravenous access placed and were cooled by the rapid intravenous infusion of 4°C cold normal saline. The recommended dose of coolant was 15 to 20 mL/kg. During transport, patients were monitored as usual (continuous) electrocardiogram, heart rate, and peripheral oxygen saturation). On arrival at the hospital, vital signs, including body temperature, were reassessed. Afterwards, in-hospital intensive care therapy, including TH, urgent myocardial revascularization (if indicated), goal-directed hemodynamic support, and control of blood glucose, ventilation, and seizures as described by Sunde and colleagues [9], was performed in all ICUs.

Cold saline was stored in the refrigerator of every ambulance and packed in bags of 250 or 500 mL. Body temperature was measured tympanally. Every measurement was repeated thrice and averaged for further analysis. During the transport, midazolam and fentanyl or sufentanyl were used for sedation and analgesia, and pipecuronium was administered for muscle paralysis. When necessary, continual infusion of noradrenaline or dopamine was used for circulatory support.

All treatment decisions and interventions in the field were made by emergency physicians only.

The control group patients were resuscitated in the 1-year period before the study was initiated and were selected consecutively from the health documentation of both administrative regions. The same inclusion and exclusion criteria as in the TH group patients were retrospectively applied to minimize selection bias. The control group patients underwent a standard process of CPR and pre-hospital and in-hospital care according to the guidelines, including in-hospital therapeutic hypothermia, but did not undergo any pre-hospital cooling attempt [1].

All resuscitation details and characteristics of the pre-hospital and in-hospital courses were recorded in Utstein style [10]. Classification of the different types of causes of cardiac arrest followed the European Resuscitation Council Guidelines for Resuscitation (2005) and European Society of Cardiology guidelines [1,11,12]. The definite identification of cardiac arrest was based on the individual assessment of the medical history, prodromal pre-arrest symptoms, pre-hospital clinical examination (including 12-lead electrocardiogram), in-hospital course of the disease, and the autopsy results, if indicated.

### Outcome assessment

The primary endpoint was a decrease of tympanic temperature (TT) from baseline to hospital admission. Secondary endpoints were the possibility of achieving a TT of not more than 34°C on hospital admission, the pre-hospital (and early in-hospital, respectively) incidence of the post-resuscitation adverse events, and the presence of a favorable neurological outcome at hospital discharge. Monitored adverse events of cooling were the new onset of pulmonary edema during transport and within 12 hours after admission, bradycardia, non-sustained ventricular tachycardia/fibrillation, recurrence of cardiac arrest and ongoing CPR at the hospital, and the need of vasopressoric support for hypotension during transport. The neurological outcome was assessed by the cerebral performance category (CPC) scoring system. Categories 1 and 2 were considered favorable [10].

### Statistical analysis

Mean values ± standard deviation or percentages were calculated for all variables. Differences between the groups were compared by the chi-square test. Statistical significance was calculated by the Fisher exact test for alternative variables. The statistical significance for continuous variables was determined by the Student *t *test. To analyze the impact of transport duration on the cooling efficacy, we divided the transport duration into four quartiles. Independent predictors of the presence of favorable neurological outcome at hospital discharge were evaluated by multivariate logistic regression analysis of a sample of all 80 patients from both groups together. Data were analyzed with JMP 3.2 statistical software (SAS Institute Inc., Cary, NC, USA). A *P *value of less than 0.05 was considered statistically significant.

## Results

### Baseline characteristics and demographic data

A total of 41 patients underwent baseline assessment. One of them died before any cooling attempt. Another 40 patients were cooled following the protocol (TH group). The same number of patients was included in the control group. Table 1 summarizes the baseline demographic data, and Table 2 summarizes the characteristics of cardiac arrest causes and the CPR process. In the TH group, more patients received bystander CPR.

**Table 1:** Baseline demographic variables

	TH group	Control group	*P *value
Number of patients	40	40	
Age, years	61.4 ± 18.1	61.3 ± 17.3	0.975
Males	34 (85.0)	29 (72.5)	0.274
Body weight, kg	83.6 ± 17.0	81.3 ± 18.1	0.571
Arterial hypertension	24 (60.0)	23 (57.5)	0.820
Diabetes mellitus	13 (32.5)	10 (25.0)	0.459
Active smokers	15 (37.5)	14 (35.0)	1.000
Hyperlipoproteinemia	12 (30.0)	10 (25.0)	0.616
History of myocardial infarction	17 (42.5)	13 (32.5)	0.356
History of PCI or CABG or both	10 (25.0)	8 (20.0)	0.592
Congestive heart failure	10 (25.0)	12 (30.0)	0.616
Significant valvular disease	4 (10.0)	5 (12.5)	0.723
Peripheral vascular disease	5 (12.5)	7 (17.5)	0.754
Chronic renal failure	7 (17.5)	2 (5.0)	0.077
Chronic pulmonary disease	9 (22.5)	10 (25.0)	0.793
History of endocrinous disease	2 (5.0)	2 (5.0)	1.000
History of psychiatric disorder or alcoholism	8 (20.0)	8 (20.0)	0.692

Values other than 'Number of patients' and *P *values are expressed as mean ± standard deviation or as number (percentage). CABG, coronary artery bypass graft surgery; PCI, percutaneous coronary intervention; TH, therapeutic mild hypothermia.

**Table 2:** Cardiac arrest causes, initial rhythm, and cardiopulmonary resuscitation variables

	TH group	Control group	*P *value
Causes of cardiac arrest			
STEMI	12 (30.0)	13 (32.5)	0.809
NSTEMI/unstable angina	8 (20.0)	3 (7.5)	0.104
Complication of congestive heart failure	8 (20.0)	9 (22.5)	0.785
Pulmonary embolism	2 (5.0)	3 (7.5)	0.644
Metabolic	2 (5.0)	4 (10.0)	0.396
Secondary hypoxic	5 (12.5)	6 (15.0)	0.745
Unknown	3 (7.5)	2 (5.0)	0.644
Initial rhythm			
Ventricular fibrillation	21 (52.5)	18 (45.0)	0.655
Asystole	15 (37.5)	14 (35.0)	1.000
Pulseless electrical activity	4 (10.0)	8 (20.0)	0.348
CPR variables			
Time from collapse to any resuscitation attempt, minutes	4.0 ± 3.0	4.5 ± 3.4	0.454
Time from collapse to ROSC, minutes	26.8 ± 16.9	25.4 ± 13.9	0.695
Any bystander CPR attempt	26 (65.0)	17 (42.5)	0.043
Cumulative defibrillation energy in ventricular fibrillation patients, J	877 ± 763	1,097 ± 1,099	0.468
Cumulative epinephrine dose, mg	4.7 ± 4.5	4.5 ± 3.6	0.892
Device-based heart massage	5 (12.5)	4 (10.0)	0.723

Values other than *P *values are expressed as number (percentage) or as mean ± standard deviation. CPR, cardiopulmonary resuscitation; NSTEMI, non-ST-segment elevation myocardial infarction; ROSC, return of spontaneous circulation; STEMI, ST-segment elevation myocardial infarction; TH, therapeutic mild hypothermia.

### Cooling procedure

**Figure 1:**
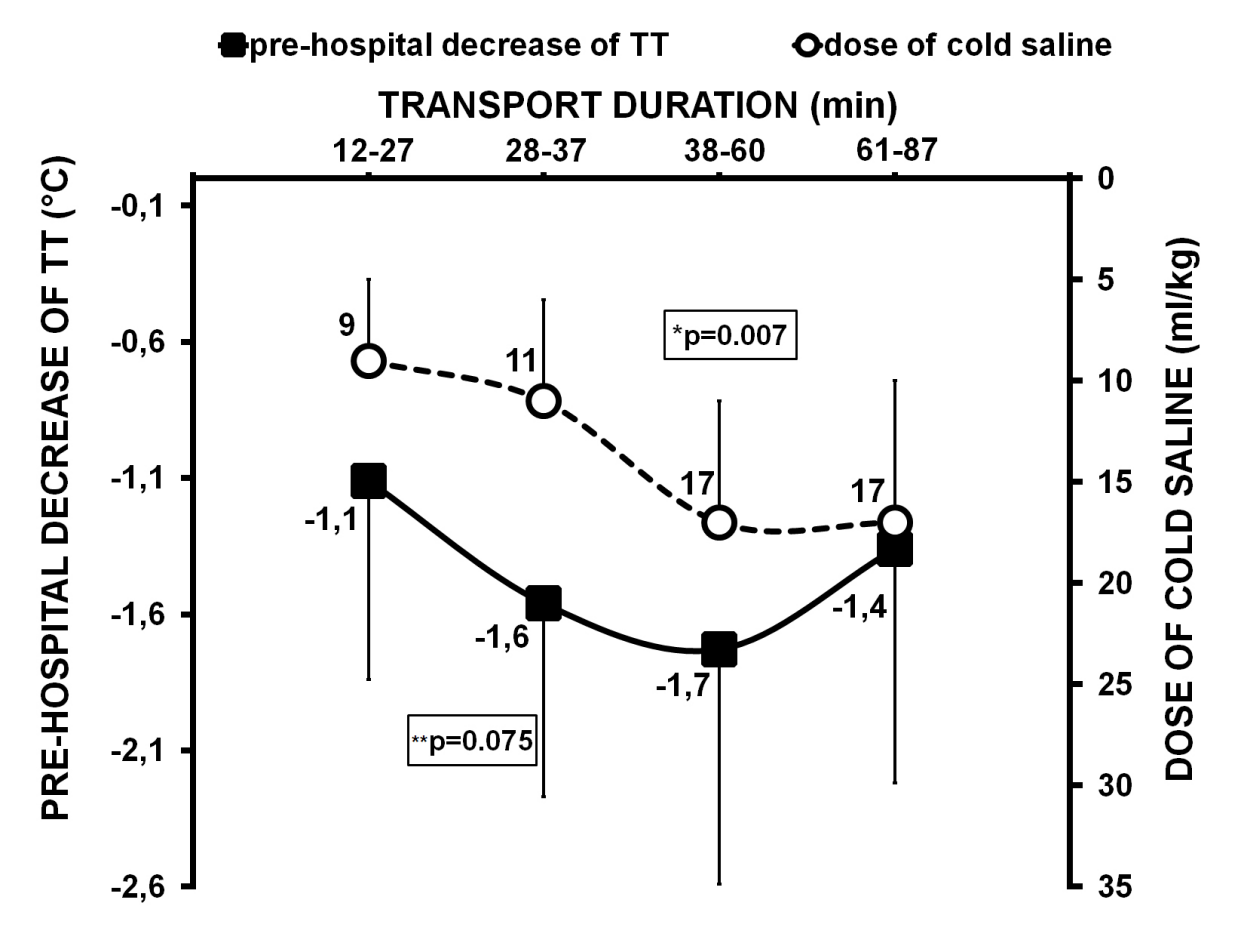
**The impact of transport duration on the cooling rate and on the decrease of tympanic temperature (TT) during transport**. *P for dose of cold saline; **P for pre-hospital TT decrease.

In the TH group, the administration of 12.6 ± 6.4 mL/kg (1,032 ± 546 mL) of 4°C normal saline led to a TT decrease of 1.4 ± 0.8°C (from 36.2 ± 1.5 to 34.7 ± 1.4°C; *P *< 0.001) in 42.8 ± 19.6 minutes. The TT decrease following the administration of at least 12.6 mL/kg of coolant was more intense than the decrease induced by a lower dose (-1.8 ± 0.7 versus -1.1 ± 0.7; *P *= 0.008). A TT of not more than 34°C was reached in 17.5% of cooled patients, and a TT of not more than 35°C was reached in 52.5% of cooled patients. The administered volume of cold saline correlated linearly with a pre-hospital decrease of TT in the TH group (*r *= -0.611, *P *< 0.001). The impact of pre-hospital transport time on the decrease of TT achieved is shown in Figure 1. The most effective cooling was associated with a transport time of 38 to 60 minutes and with the administration of 17 mL/kg of cold saline.

There was no significant difference in time from collapse to hospital arrival between the groups (TH group: 59.6 ± 29.5 minutes, control group: 61.6 ± 23.8 minutes; *P *= 0.746). Underdosing of coolant was observed in 23 (57.5%) patients in the TH group. The most frequent reported cause was a short pre-hospital transport time (73.9%). In the two groups, we observed a comparable incidence of post-resuscitation adverse events (Table 3). In the TH group, a trend toward a lower need of catecholamines during transport was detected (Table 3).

**Table 3:** The pre-hospital incidence of post-resuscitation adverse events

	TH group, number (percentage)	Control group, number (percentage)	*P *value
Bradycardia	1 (2.5)	1 (2.5)	1.000
Non-sustained ventricular fibrillation/tachycardia	2 (5.0)	1 (2.5)	0.541
Recurrence of cardiac arrest	4 (10.0)	5 (12.5)	0.723
Requirement of vasopressors during transport	14 (35.0)	21 (52.5)	0.115
New pulmonary edema during transport and in 12 hours after admission	0 (0)	1 (2.5)	0.314
Ongoing CPR at hospital arrival	3 (7.5)	5 (12.5)	0.456

CPR, cardiopulmonary resuscitation; TH, therapeutic mild hypothermia.

### In-hospital therapy and neurological outcome

Patients in the two groups did not differ in the in-hospital markers of the severity of post-cardiac arrest syndrome, in the intensity of organ-supporting therapy, or in the number of patients treated by in-hospital TH (Table 4). The majority of patients in both groups underwent in-hospital cooling (TH group: 85.0%, control group: 80.0%; *P *= 0.556).

**Table 4:** In-hospital course of the post-resuscitation disease and neurological outcome

	TH group	Control group	*P *value
Number of days on mechanical ventilation	11.4 ± 16.4	14.3 ± 23.4	0.531
Number of days of ICU stay	16.0 ± 17.9	18.7 ± 27.1	0.596
Number of post-resuscitation organ dysfunctions	1.4 ± 1.4	1.3 ± 1.3	0.633
Major bleeding	3 (7.5)	6 (15.0)	0.288
Infection	19 (47.5)	17 (42.5)	0.653
Urgent coronary angiography	25 (62.5)	17 (42.5)	0.073
Direct PCI/CABG	14 (35.0)	14 (35.0)	0.813
Systemic thrombolysis	0 (0)	3 (7.5)	0.488
Intra-aortic balloon pump	4 (10.0)	4 (10.0)	1.000
Continual renal replacement method	2 (5.0)	3 (7.5)	0.644
CPC 1 or 2 at discharge	18 (45.0)	11 (27.5)	0.103
In-hospital mortality	15 (37.5)	22 (55.0)	0.116

Values other than *P *values are expressed as mean ± standard deviation or as number (percentage). CPC, cerebral performance category; ICU, intensive care unit; PCI/CABG, percutaneous coronary intervention/coronary artery bypass graft; TH, therapeutic mild hypothermia.

In the TH group, there were trends to higher incidence of a favorable neurological outcome at hospital discharge and to lower in-hospital mortality than in the control group (Table 4). Providing of bystander CPR was associated with a trend to improved incidence of a favorable neurological outcome in the TH group (bystander CPR: 53.8%, no bystander CPR: 28.6%; *P *= 0.125) but not in the control group (bystander CPR: 23.5%, no bystander CPR: 30.4%; *P *= 0.629).

The coupling of pre-hospital hypothermia with subsequent in-hospital cooling was associated with the higher frequency of favourable neurological outcome at hospital discharge than other treatment (in-hospital or pre-hospital cooling only or no TH) (52.9% versus 23.9%; *P *= 0.008) throughout the whole sample of all 80 patients. Multivariate analysis confirmed the predictive value of the coupled approach for a favourable neurological outcome at discharge (odds ratio [OR] 4.1, 95% confidence interval [CI] 1.1 to 18.2; *P *= 0.046). The other significant positive predictor was the presence of ventricular fibrillation as the initial rhythm (OR 4.26, 95% CI 1.1 to 18; *P *= 0.039) and the negative predictor was time from collapse to ROSC of more than 22 minutes (OR 0.21, 95% CI 0.05 to 0.71; *P *= 0.019). Other parameters such as age, medical history of diabetes mellitus, cause of OHCA, time from ROSC to hospital arrival, providing of bystander CPR, recurrence of cardiac arrest, and the need for use of catecholamines during transport did not reach significant value.

## Discussion

The main finding of our analysis is that pre-hospital induction of TH by RIVA in successfully resuscitated OHCA patients led to a significant TT decrease despite relatively low doses of cold normal saline. The positive impact of TH on the prognosis of OHCA survivors has been shown by several studies [13-16]. In accordance with the recommendation of initiating TH as soon as possible, moving of the first cooling attempt to the pre-hospital phase seems to be a step leading to very early intervention. Evidence of further prognosis improvement by pre-hospital cooling has not been shown yet. However, a battery of other arguments, particularly the pathophysiological principle of cerebral ischemia-reperfusion injury per se and the results of animal experiments and of some clinical studies in humans, favor this approach [17-21]. Recently, Castrén and colleagues [22] reported the results of the clinical study PRINCE (Pre-Resuscitation Intra-Nasal Cooling Effectiveness), which showed in the subgroup analysis the improvement of prognosis of OHCA patients by pre-hospital intranasal intra-arrest cooling.

Mostly, the studies analyzed the pre-hospital induction of TH by the RIVA technique [2-5].

Virkkunen and colleagues [2] cooled 13 pre-hospital cardiac arrest patients by the rapid administration of 30 mL/kg of ice-cold Ringer's solution. The authors achieved a pre-hospital decrease of esophageal temperature of 1.8°C [2]. In a later study, Kim and colleagues [3] randomly assigned 125 patients to receive standard care with or without intravenous cooling pre-hospitally (500 to 2,000 mL of 4°C normal saline). In the hypothermia group, 87% of patients were cooled, and they achieved a clinically relevant pre-hospital decrease of esophageal temperature when compared with the control group (1.24 ± 1.09°C versus 0.10 ± 0.94°C; *P *< 0.001). Pre-hospital cooling was safe and was associated with a trend toward improved survival of patients who received cooled ventricular fibrillation prior to hospital arrival [3]. Recently, Kämäräinen and colleagues [4] randomly assigned 37 patients to pre-hospital cooling or standard care. The administration of 27 mL/kg of cold normal saline to 19 patients led to a decrease in nasopharyngeal temperature of 1.5 ± 0.8°C, whereas 18 patients in the control group did not exhibit any temperature change (0.1 ± 0.6°C; *P *< 0.001). A high proportion of patients with a favorable neurological outcome at discharge was observed (42% versus 44%; *P *> 0.05) [4]. Finally, Hammer and colleagues [5] reported the results of a French study. From a total of 99 patients, 22 were cooled by RIVA prior to hospital arrival and 9 (41%) of them reached a body temperature of less than 35°C. The remaining 77 patients underwent a standard treatment and 14 (18%) of them experienced a temperature drop to less than 35°C [5]. The studies, though not designed primarily for analysis of neurological outcome, clearly demonstrated that pre-hospital applying of large amount of cold crystalloids is a safe and effective procedure and that without an active cooling approach, no significant spontaneous cooling occurs.

In our study, although the dose of coolant was considerably smaller than those in the studies by Kämäräinen and colleagues and Virkkunen and colleagues, we observed a significant and clinically relevant pre-hospital decrease of TT. This decrease was similar to the one reported by Kim and colleagues, who observed a significant decrease of esophageal temperature in those patients who were administered both a full dose of 2,000 mL of normal saline and a dose of between 500 and 2,000 mL. However, even the correlation of the administered coolant dose and reached TT decrease was linear in our study, we want to stress that cooling effectiveness depends not only on the dose of coolant but also on other variables: First, cooling effectiveness depends on the air temperature inside the ambulance and the infusion bags' temperature stability, which is defined particularly by the initial infusion temperature, the type of infusion package, the initial volume of the infusion bag, and the infusion rate. Second, a period from the completion of cooling until hospital arrival determines a time period for potential unintentional rewarming of the patient. Third, the patient's cooling responsiveness, which is related mainly to the suppression of shivering, can influence cooling effectivity. Finally, the method of measuring body temperature may be important. In our study, infusion bags of no larger than 250 or 500 mL were used. We can speculate that rapid and repeated application of small-volume bags may reduce a spontaneous infusion rewarming during its administration and may enhance the cooling potency. We consider that this calls for further studies to optimize the pre-hospital RIVA cooling procedure.

Moreover, our analysis showed that the decrease of TT during pre-hospital transport followed the J curve. The most intense TT decrease was associated with a transport time of 38 to 60 minutes. Longer transport time was not associated with the enhancement of cooling efficacy, and a trend to rewarming was found. Previously, Kliegel and colleagues [23] demonstrated that cold infusion alone fails to keep patients cool. Thus, we propose that in the case of a transport time of more than 45 minutes, the second reduced bolus of cold infusion be considered.

An important issue is procedural safety. Neither in previous studies nor in our study was a higher incidence of early post-resuscitation adverse events observed in the cooling group. It is noteworthy that a trend toward a lower frequency of the need for vasopressoric support was recognized in the TH group. Kim and colleagues [3] described a similar effect, and Kämäräinen and colleagues [4] described the opposite. We can speculate that volume expansion with normal saline can contribute to hemodynamic stabilization in some patients. Previously, we showed that fluid responsiveness of cardiac arrest survivors with a low cardiac output is high in general [24]. In addition to the patient's clinical condition, the hemodynamic effect of the coolant is probably related to the dose and infusion rate. Thus, it is possible that a dose of 10 to 20 mL/kg is more hemodynamically suitable than 30 mL/kg.

The impact of the procedure on the clinical outcome was also analyzed. The neurological outcome at hospital discharge in the TH group reflects the results of the trials analyzing in-hospital and pre-hospital hypothermia [4,9,25]. Bystander CPR was provided more frequently in the TH group. Perhaps surprisingly, bystander CPR was not associated with a clear improvement of the neurological prognosis. A similar observation was reported by Kämäräinen and colleagues [4]. The main reason for this observation, in our view, is that providing bystander CPR is the primary factor that determines whether ROSC is achieved. In our study, we included only successfully resuscitated OHCA patients. In this selected group, the subsequent impact of bystander CPR on the neurological outcome may be less intense, probably demonstrable only by larger sample size.

Because not all patients in the two groups were treated by in-hospital hypothermia, the whole sample of 80 patients can be distributed into four subgroups according to the performed cooling schedule: pre-hospital cooling followed by in-hospital TH, in-hospital cooling only, pre-hospital cooling only, and no administration of TH. We believe that this distribution reflects real-life practice and that is why we calculated the odds for the first-mentioned approach. Despite the study limitations (described below), the close coupling of pre-hospital TH induction with its in-hospital continuation predicted a favorable neurological outcome. We stress that this coupling is not definite evidence of a benefit from pre-hospital cooling. However, it suggests that early pre-hospital TH induction closely followed by the sophisticated in-hospital intensive care, including TH, can help improve further prognosis. In any case, more studies are required. In the future, a comparison of the RIVA method with other pre-hospital cooling techniques like surface cooling (as demonstrated by Uray and colleagues [26]) or even an investigation of a combinatory pre-hospital cooling approach would be beneficial.

There are some limitations to our study. First, the study was not randomized. Second, body core temperature was measured by one method (measurement was semi-continual and tympanal). Third, in-hospital intensive care was not controlled by the study protocol. Fourth, some outcomes of the study are influenced by longer pre-hospital times, reflecting local protocols and the availability of hospitals with a catheterization laboratory. Fifth, experiences from the physician-staffed EMS may not be completely applicable by the other types of EMS.

## Conclusions

Pre-hospital induction of TH by the RIVA method has been shown to be efficient, even with the lower dose of coolant as was investigated in previous studies. This dose can be associated with the favorable impact on the circulatory stability early after the ROSC and, when followed by in-hospital TH, can potentially improve the prognosis of the patients. We call for further studies to optimize pre-hospital cooling by cold crystalloids for the optimal cooling efficacy along with the beneficial effect on hemodynamics.

## Key messages

• Pre-hospital induction of therapeutic hypothermia by a rapid intravenous administration of 4°C cold normal saline can be effective, even with a dose of about 15 mL/kg.

• Volume expansion associated with intravenous cooling can contribute to hemodynamic stabilization.

• The close coupling of pre-hospital induction of therapeutic hypothermia with its in-hospital continuation can help to improve the prognosis of patients.

## Abbreviations

CI: confidence interval; CPR: cardiopulmonary resuscitation; EMS: emergency medical service; OHCA: out-of-hospital cardiac arrest; OR: odds ratio; RIVA: rapid intravenous administration of cold crystalloids; ROSC: return of spontaneous circulation; TH: therapeutic mild hypothermia; TT: tympanic temperature.

## Competing interests

The authors declare that they have no competing interests.

## Authors' contributions

RŠ and AT helped to design the study, were the main investigators of the study, shared responsibility for the pre-hospital recruitment of the patients, and supervised the analysis and analyzed all data. JŠ helped to design the study and shared responsibility for the pre-hospital recruitment of the patients. VČ helped to design the study and shared responsibility for coordination of the in-hospital care. PD shared responsibility for coordination of the in-hospital care. All authors were involved in the collection of all data, drafted and revised the manuscript, and read and approved the final manuscript.

## Authors' information

JŠ is a head of the Czech Society of Emergency and Disaster Medicine. VČ is a national representative of the European Society of Intensive Care Medicine, a head of the Czech Society of Intensive Care Medicine, a member of the committee of the Czech Society of Anaesthesiology and Intensive Care Medicine, and a head of the Department of Anesthesiology and Intensive Care, Charles University in Prague, Faculty of Medicine in Hradec Kralove, Czech Republic.
